# Ultrasound Guidance for Vaginal Drainage of Postoperative Pelvic Hematoma: A Case Report

**DOI:** 10.1155/S1064744994000281

**Published:** 1994

**Authors:** Thomas E. Snyder, Sebastian Faro

**Affiliations:** Department of Gynecology and Obstetrics University of Kansas School of Medicine 3901 Rainbow Boulevard Kansas City KS 66160-7316 USA

## Abstract

*Background:* Postoperative pelvic fluid collection is almost a universal consequence of gynecologic surgery. At one end of the spectrum are minimal collections of peritoneal fluid or blood that are clinically inconsequential. At the other end are hematomas and abscesses requiring active intervention for the patient to recover.

*Case:* This case report describes a symptomatic vaginal vault hematoma occurring after vaginal hysterectomy. Ultrasonography was used to accurately identify the hematoma and guide intraoperative drainage. The patient fully recovered without complication.

*Conclusion:* Accurate visual guidance of instrumentation to decompress postoperative retroperitoneal hematomas is a marked improvement over non-visual techniques utilizing palpation only. The hematoma cavity can be more easily entered and the hematoma or abscess more completely drained, expediting the recovery of the patient affected by this problem.


Infectious Diseases in Obstetrics and Gynecology 1:293–297 (1994)
© 1994 Wiley-Liss, Inc.

### Ultrasound Guidance for Vaginal Drainage of Postoperative Pelvic Hematoma: A Case Report

**Thomas E. Snyder and Sebastian Faro**

*Department of Gynecology and Obstetrics, University of Kansas School of Medicine, Kansas City, KS*

ABSTRACT

***Background:*** **Postoperative pelvic fluid collection is almost a universal consequence of gynecologic surgery. At one end of the spectrum are minimal collections of peritoneal fluid or blood that are clinically inconsequential. At the other end are hematomas and abscesses requiring active intervention for the patient to recover.**

***Case:*** **This case report describes a symptomatic vaginal vault hematoma occurring after vaginal hysterectomy. Ultrasonography was used to accurately identify the hematoma and guide intraoperative drainage. The patient fully recovered without complication.**

***Conclusion:*** **Accurate visual guidance of instrumentation to decompress postoperative retroperitoneal hematomas is a marked improvement over non-visual techniques utilizing palpation only. The hematoma cavity can be more easily entered and the hematoma or abscess more completely drained, expediting the recovery of the patient affected by this problem.** © 1994 Wiley-Liss, Inc.

KEY WORDS

Postoperative fluid collection, pelvic abscess, vaginal hysterectomy

Hematoma or abscess formation is a relatively common complication of vaginal or abdominal hysterectomy. Prior to the advent of computerized tomography (CT) and ultrasonography, drainage of these collections occurred spontaneously through the rectum or vagina, or manual drainage transvaginally was performed based on physical examination and palpation. The obvious complications of these procedures included inadvertent bladder, bowel, or peritoneal cavity entry. In addition, the hematoma or abscess may be inadequately evacuated secondary to multiple loculations and/or separate fluid collections in the pelvis. The utilization of modern ultrasonography or CT-guided drainage of postoperative pelvic hematomas offers a distinct advantage over undirected drainage. A case is described utilizing real-time ultrasonography to effectively evacuate bilateral 6-cm postoperative pelvic hematomas following vaginal hysterectomy and anterior and posterior repair.

### CASE REPORT

A 35-year-old female, $G_3P_3$ presented with classic symptoms of second-degree uterine descensus and cystorectocele of longer than 5 years duration, beginning shortly after the delivery of her second child. Symptomatology included pelvic discomfort on prolonged standing and visual protrusion of pelvic structures which increased during the 1.5 years following her third delivery and resulted in her clinical presentation. Her medical history was unremarkable except for a cervical lymph node biopsy at age 13 years diagnosed as Hodgkin's disease. However, the diagnosis was revised following splenectomy and exploratory laparotomy to mononucleosis. The patient denied any other surgery or

**Address correspondence/reprint requests to Dr. Thomas E. Snyder, Department of Gynecology and Obstetrics, University of Kansas School of Medicine, 3901 Rainbow Boulevard, Kansas City, KS 66160-7316.**

Received October 18, 1993
Accepted February 4, 1994

significant hospitalization. Her gynecologic history was unremarkable except for the noted anatomic defects. Despite a marked cystorectocele, the patient described no symptoms of stress urinary incontinence or rectal splinting at the time of defecation.

The patient entered the University of Kansas Medical Center on the day of surgery for a vaginal hysterectomy and anterior and posterior repair. The operation was performed without incident with blood loss of 310 cc. She remained quite stable until approximately 6 h postoperatively when she began to report inadequate pain control. In addition, mild hypotension (100/50), tachycardia (120/min), and oliguria (<30 cc/h) during the previous 30 min were noted. The physical examination revealed minimum abdominal findings. The pelvic examination suggested a retroperitoneal hematoma at the vaginal apex. The initial hemoglobin was 9.4 g/dl, consistent with surgical blood loss. Therefore, pain control was obtained and fluid balance corrected with resolution of her hypotension and tachycardia. A follow-up examination revealed a gradual decrease in hemoglobin to 6.8 g/dl over 12 h. The patient was transfused with 2 units of packed red blood cells and remained quite stable with hemoglobin values subsequently remaining at 9–10 g/dl. She was initially given cetoxitin sodium (Mefoxin, Merck, Sharp & Dohme, West Point, PA) prophylaxis at the time of surgery and subsequently was maintained on this antibiotic following the diagnosis of postoperative hematoma. She remained afebrile without other complaints throughout the initial hospitalization and was discharged home on postoperative day 5.

The patient reentered the hospital on postoperative day 14 complaining of 2 days of mild vaginal bleeding. While she remained afebrile during her home follow-up, her white blood cell (WBC) count was noted to have increased from 14,000 to 19,000 during the 5 days preceding her readmission to the hospital. (A WBC count of 12,000–14,000/cc is considered normal following splenectomy.) A pelvic ultrasound revealed bilateral retroperitoneal hematomas, 4 cm in diameter, at the vaginal apex, one of which was spontaneously draining. Broad-spectrum antibiotics were initiated with ampicillin sodium/sulbactum sodium (Unasyn, Roerig, New York, NY) and gentamycin (Garamycin, Schering, Kenilworth, NJ).

On the following day, she was taken to the operating room where ultrasonography was used to guide drainage of both hematomas and placement of a suction catheter. The postoperative course was unremarkable, requiring no further transfusion or change in antibiotics. A culture of the hematoma fluid revealed moderate growth of a gram-positive coccus consistent with *Streptococcus* and *Enterococcus* species.

### DISCUSSION

Pelvic hematoma or abscess is a common complication of gynecologic surgery. Surgical drainage of a pelvic abscess was first performed by Recamier (1830–1840).[1] Prior to widespread use of antibiotics, resolution of these masses was obtained by spontaneous or purposeful transvaginal or transrectal drainage. Spontaneous intraabdominal rupture occasionally occurred with attendant marked morbidity and mortality. Prior to the antibiotic era, this event resulted in almost 100% mortality, as reported by Pedowitz and Bloomfield[2] in a review of 143 cases treated prior to 1947. Prior to 1960, authors continued to report high mortality rates even with aggressive surgical and antibiotic therapy.[3] In the 1970s, colpotomy incision of the cul-de-sac of Douglas was utilized for drainage of these abscesses, as exemplified by the report of Rubenstein and Mishell.[4] This approach is limited by the requirement of a mass reasonably accessible through the vaginal apex, i.e., a mass dissecting the retrovaginal septum or retroperitoneal space at the cul-de-sac. These authors found that, while good drainage of the cul-de-sac was effected, complete drainage of the pelvic abscess was not assured, e.g., in the presence of multiple fluid loculations. Greater than one third of the patients thus treated required additional major surgical procedures because of continued infection, pain, or other complications. Rivlan[5,6] subsequently produced 2 reviews, one of a series of 348 patients treated in this fashion with a 6.5% diffuse sepsis rate and the other of a separate group of 59 patients with 2 reported deaths. In addition, further surgery was required during the same or a subsequent admission in 20% of the patients and at a later admission in 18% of the patients.[5,6] Indeed, well-known authors as recently as 1985 have decried the use of colpotomy for treatment of tuboovarian abscesses.[7]

With the advent of improved CT imaging in the

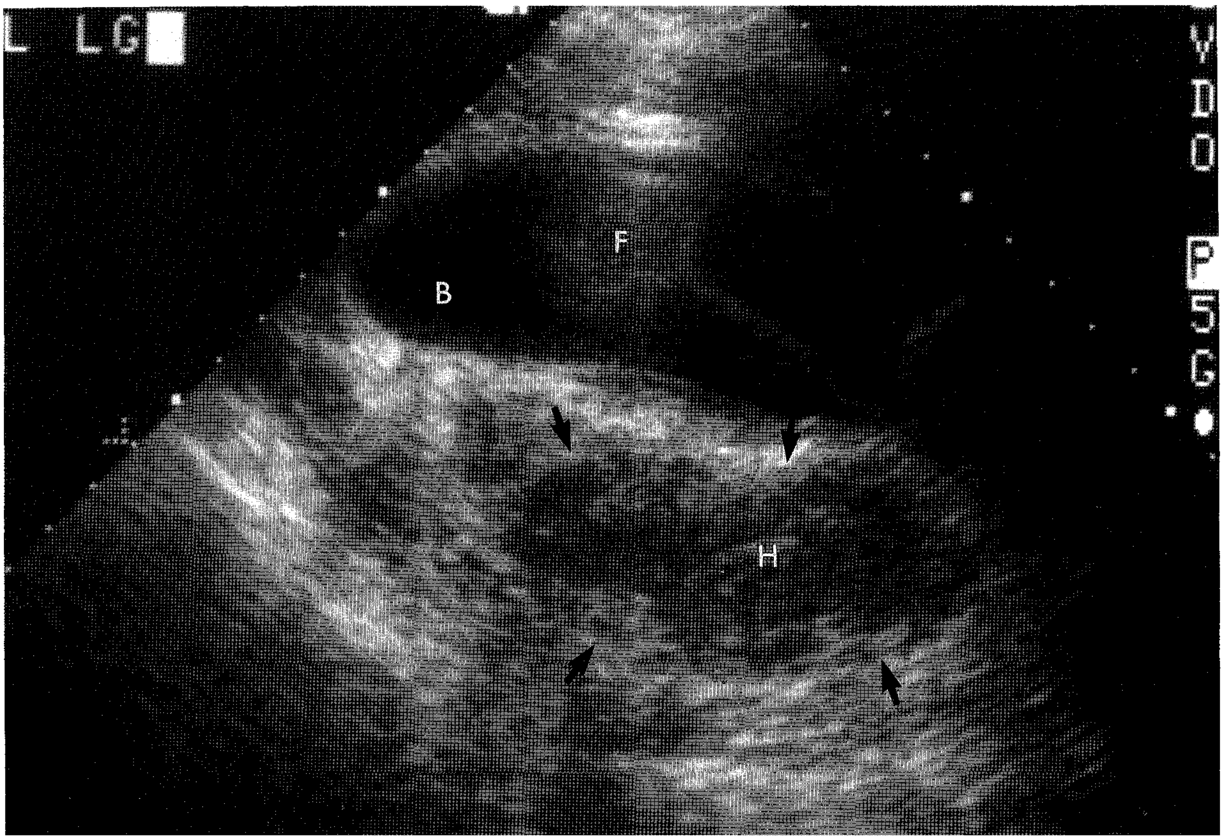

**Fig. 1. Longitudinal scan of the lower abdomen and pelvis. Bladder (B) is distended with Foley catheter (F). Hematoma (H, arrows) approximately 6 cm in diameter is posterior to the bladder.**

1980s, transabdominal techniques have been used for drainage of spontaneous and postoperative intraabdominal abscesses and hematomas.[8–10] Since its description by Smith and Bartrum[11] in 1974, ultrasonography has also been successfully used for transabdominal drainage of abscesses. Multiple reports have been produced in the interim, and many authors consider ultrasonography to be the method of choice.[12] The criteria for abdominal ultrasound-guided aspiration of a pelvic abscess include the presence of a well-defined unilocular abscess cavity, a safe drainage route, evaluations by surgery and radiology services, and the immediate availability of operative intervention.[12–14] Indeed, Nosher and co-workers[13] advocate drainage of all abdominal and pelvic abscesses by the percutaneous route. On the other hand, laparoscopic inspection, dissection, and drainage of tuboovarian abscesses from pelvic inflammatory disease or postsurgical hematomas have been recently used with some success.[15]

Review of the gynecologic literature over the past 10 years reveals a paucity of reports on ultrasound-guided vaginal drainage of pelvic hematomas or abscesses. There are multiple reports available in the literature of transvaginal ultrasound procedures, especially follicle aspiration,[16,17] and ultrasound-guided aspiration of ectopic pregnancy.[18] Therefore, it is surprising that more literature regarding vaginal procedures is not available. Vaginal decompression of a pelvic abscess is hampered by the proximity of the bladder, bowel, vasculature, and other adnexal structures. In these situations, transrectal ultrasound guidance of drainage has been described.[19,20] The procedure is advocated in men and nulliparous women in whom transvaginal access is not an option. A novel transgluteal approach has been reported by Butch et al.,[21] although a significant amount of soft tissue must be transversed to reach the abscess or hematoma. Haji et al.,[22] writing in 1988, still advocated laparotomy drainage of tuboovarian masses, emphasizing

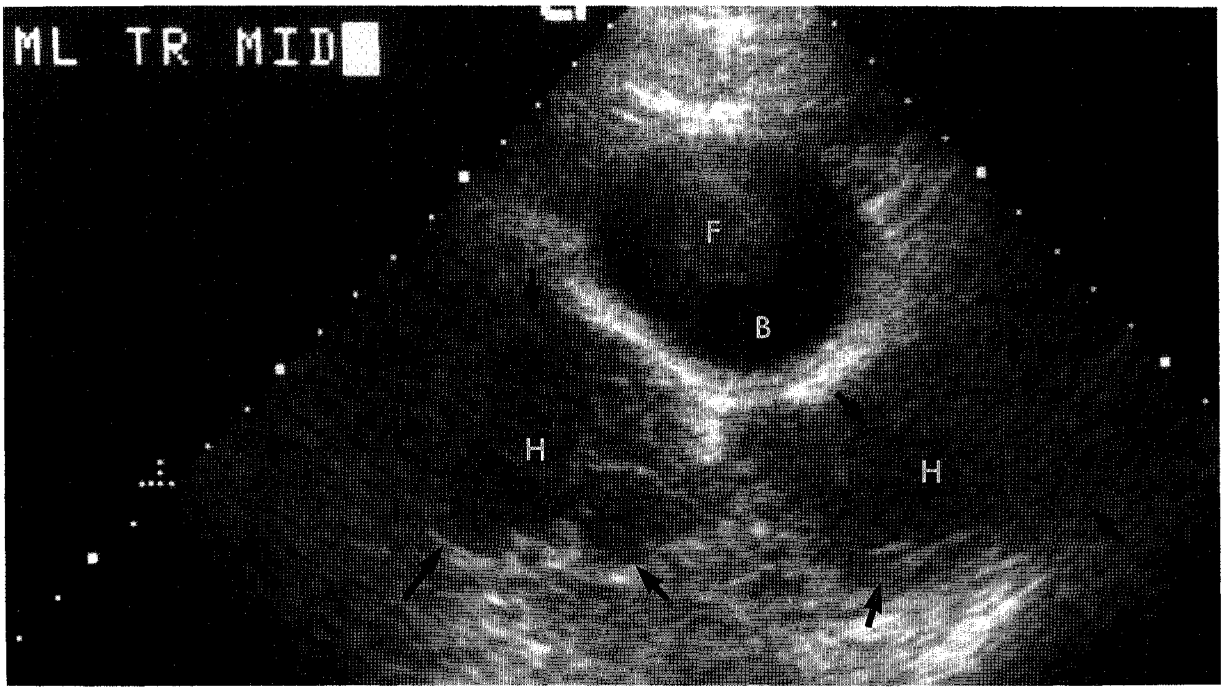

Fig. 2. Transverse scan of the lower abdomen and pelvis. Bladder (B) with Foley catheter (F) within. Hematoma (H, arrows) is posterior to the bladder.

that any attempt at vaginal drainage is hazardous and could be associated with injury to the interposed bowel. However, these authors did advocate vaginal drainage of rectovaginal and vaginal apex abscesses. Ultrasonography was found to be valuable in following the resolution of these masses. On the other hand, recent literature is available to advocate ultrasound-guided transvaginal drainage of posthysterectomy abscesses.[13,23] McArdle et al.[23] demonstrated good visualization and drainage of a pelvic abscess and placement of a Foley catheter. In addition, the bowel, bladder, and peritoneal cavity were adequately seen, and entry into these structures was thus avoided.

In the current case, abdominal ultrasonography was carried out after standard preparation and draping of the patient for a vaginal approach to the bilateral hematomas. The sterile technique was not compromised. Initially, the bladder and the bilateral retroperitoneal hematomas were identified just off the midline. Figure 1 demonstrates a longitudinal scan of the lower abdomen and pelvis. The bladder, labeled "B," is distended and a Foley catheter, labeled "F," is identified within it. Posterior to the bladder is a lobulated soft-tissue mass, approximately 6 cm in diameter (indicated by arrows and labeled "H"). Figure 2 demonstrates a transverse scan of the same area. The urinary bladder with a Foley catheter within it is labeled "B." Inferior to the bladder, a bilocular cystic and solid mass, approximately 5 cm in diameter, is indicated by the arrows and labeled "H." A large-gauge intercath was inserted into both hematomas, with evacuation of old blood which was submitted for culture. Ultrasonography allowed direct visualization of intercath placement and irrigation of the hematoma cavity. Subsequently, the opening into the left hematoma cavity was enlarged and a suction drain was placed, again with ultrasound guidance to insure proper placement.

This case demonstrates two principles in the management of postoperative pelvic hematomas. First, antibiotic therapy should be broad spectrum, offering coverage for the mixed bacterial flora commonly found in these masses. The use of Mefoxin or other broad-spectrum cephalosporins may allow overgrowth of *Enterococcus* or other resistant organisms, so the use of a broad-spectrum penicillin may be more useful in the non-allergic patient, especially in a patient who has received a cephalosporin for prophylaxis and then receives a cephalosporin for treatment. If the patient is allergic, the combination of vancomycin with an aminoglycoside is necessary to provide coverage against enterococci.

Second, while drainage of pelvic abscesses is a long-established mode of therapy and indeed necessary for postoperative treatment, the route of drainage remains controversial. Some authors still advocate laparotomy drainage of all hematomas and abscesses to avoid bowel, vascular, or further soft-tissue injury. This approach obviously requires another major incision, probable secondary closure of a potentially infected incision, and the risk of intraabdominal spill of grossly infected material. All of these factors extend the postoperative hospital stay and may subject the patient to significant morbidity or mortality. In selected cases, especially in cases of vaginal apex masses, real-time ultrasound-guided drainage may be preferable. This technique results in good visualization for guidance of instruments into the abscess cavity and evacuation of the contents of the hematoma or abscess. Irrigation of closed-space abscesses with saline or antibiotic solution and subsequent placement of a closed or an open drainage system to ensure continued drainage of the abscess cavity may be effected with minimal operative intervention and hospital stay.

In summary, ultrasound-guided decompression or drainage is a valuable adjunct in selected cases of postoperative pelvic hematomas or abscesses. This procedure expedites the drainage of a hematoma or an abscess and may minimize further postoperative morbidity compared with that required with conventional techniques.

### REFERENCES

1. Benigno BB: Medical and surgical management of the pelvic abscess. Clin Obstet Gynecol 24:1187–1197, 1981.
2. Pedowitz P, Bloomfield RD: Ruptured adnexal abscess (tuboovarian) with generalized peritonitis. Am J Obstet Gynecol 88:721–729, 1964.
3. Collins CG, Jansen FW: Treatment of pelvic abscess. Clin Obstet Gynecol 2:512, 1959.
4. Rubenstein PR, Mishell DR Jr: Colpotomy drainage of pelvic abscess. Obstet Gynecol 48:142–145, 1976.
5. Rivlin ME, Golan A, Darling MR: Diffuse peritoneal sepsis associated with colpotomy drainage of pelvic abscess. J Reprod Med 27:406–410, 1982.
6. Rivlin ME: Clinical outcome following vaginal drainage of pelvic abscess. Obstet Gynecol 61:169–173, 1983.
7. Landers DV, Sweet RL: Current trends in the diagnosis and treatment of tuboovarian abscess. Am J Obstet Gynecol 151:1098–1110, 1985.
8. Gerzof S, Robbins AH, Johnson WC, Birkett DH, Nabseth DC: Percutaneous catheter drainage of abdominal abscesses. N Engl J Med 305:653–657, 1981.
9. VanSonnenberg E, Ferrucci JT, Mueller PR, Wittenberg J, Simeone JF: Percutaneous drainage of abscesses and fluid collections: Technique, results, and applications. Diag Radiol 142:1–10, 1982.
10. Gronvall S, Gammelgaard J, Haubek A, Holm HH: Drainage of abdominal abscess guided by sonography. Am J Radiol 138:527, 1982.
11. Smith EH, Bartrum RJ Jr: Ultrasonically guided percutaneous aspiration of abscesses. Am J Radiol 122:308–312, 1974.
12. Gerzof SG, Robbins AH, Johnson WC, Birkett DH, Nabseth DC: Percutaneous catheter drainage of abdominal abscesses: A five-year experience. N Engl J Med 305:653–657, 1981.
13. Nosher JL, Winchman HK, Needel GS: Transvaginal pelvic abscess drainage with US guidance. Radiology 165:872–873, 1987.
14. Gerzof SG, Johnson WC: Radiologic aspects of diagnosis and treatment of abdominal abscesses. Surg Clin North Am 64:53–55, 1984.
15. Henry-Suchet J, Soler A, Loffredo V: Laparoscopic treatment of tuboovarian abscesses. J Reprod Med 29:579–582, 1984.
16. Gonen Y, Blanker J, Casper RF: Transvaginal ultrasonically guided follicular aspiration: A comparative study with laparoscopically guided follicular aspiration. J Clin Ultrasound 18:257–261, 1990.
17. Feichtinger W, Kemeter P: Transvaginal sector scan sonography for needle guided transvaginal follicle aspiration and other applications in gynecologic routine and research. Fertil Steril 45:722–725, 1986.
18. Feichtinger W, Kemeter P: Conservative treatment of ectopic pregnancy by transvaginal aspiration under sonographic control and methotrexate injection. Lancet 1:381–382, 1987.
19. Mauro MA, Jacques PF, Maudell VS, Mandel SR: Pelvic abscess drainage by the transrectal catheter approach in men. Am J Radiol 144:477–479, 1985.
20. Nosher JL, Winchman HK, Needell GS: Transrectal pelvic abscess drainage with sonographic guidance. Am J Radiol 146:1047–1048, 1986.
21. Butch RJ, Mueller PR, Ferrucci JT Jr, Wittenberg J, Simeone JF, White EM, Brown AS: Drainage of pelvic abscesses through the greater sciatic foramen. Radiology 158:487–491,1986.
22. Haji SN, Mercer LJ, Ismail MA: Surgical approaches to pelvic infections in women. J Reprod Med 33:159–163, 1988.
23. McArdle CR, Simon L, Kiejna C: Vaginal drainage of posthysterectomy abscess under direct ultrasonic guidance. Obstet Gynecol 63:90S–92S, 1984.
